# The Progress of the Sanatorium Idea

**Published:** 1912-05-18

**Authors:** 


### The Progress of the Sanatorium Idea.

The National Sanatorium Association, of which H.R.H. Princess Christian is President, held its annual general meeting on Saturday, May 11. The chair was taken at 5 p.m. by the Chairman of the Association, Mr. C. H. Garland.

After an existence of a comparatively few years, and after being engaged in practical work for five years, Mr. Garland said that they had been able to see the principles upon which their organisation was based adopted by his Majesty's Government, and put into operation by means of enormous machinery which could not fail to produce marked results in the near future. Although their Association had only been at work for so short a time, it had undoubtedly made history, and altered the face of the anti-consumption crusade in Great Britain. The educational work performed by the Association had familiarised the working-classes with the sanatorium idea. As to the relative value of the various methods which were being employed for the treatment of consumption, all they sought to do was to restore the economic value of the afflicted working-man for a sufficiently long period to make his treatment a paying proposition for the friendly society in which he was insured, and to give aid to his family. Now that these principles were going to be applied by the Government, the treatment of consumptives was going to be a source of financial profit to the Insurance Scheme, and would assist in protecting future generations. The number of beds available at the National Benenden Sanatorium has now been increased to 105. Borough Councils, Friendly Societies, and the King Edward's Hospital Fund have taken up, for patients coming under their care, the whole of the accommodation which was available when they applied. This scheme of maintenance by yearly payments from organised societies and public bodies was quite new, said Mr. Garland, when it was adopted by them, but it had proved successful. The Association enabled the working-man, hitherto dependent on charity or the Poor Law, to maintain entirely from his own pocket and without stigma, the beds in which the sufferers from his class could be treated. This was a remarkable and unique instance of self-help against an expensive malady. During the year 1911, 280 patients had been discharged from the institution. Of these 280 patients, 91 have been discharged with their disease arrested and 137 with their condition greatly improved. Of all those who had been obtained in the first stages of their disease, 68 per cent. have been discharged with their disease arrested. The Association received these patients from among the general population with no expert sifting organisation, by means of which the bad cases could be prevented from entering the institution. With the new dispensary system which was being set up under the State Insurance Scheme it should be possible to obtain incipient invalids in a much higher proportion than at present. Mr. Garland claimed that with this aid to their work sanatoria would soon justify themselves as the most hopeful method of treatment and as one of the soundest of financial undertakings. One of the most important things to be done in order to make the sanatorium movement a success was to spread widely a knowledge of the disease and the methods for its detection and treatment. The pamphlets which they had distributed were specially designed to meet the needs of the working-classes.

Mr. A. William West was elected hon. treasurer, and Mr. F. W. Wareham and Mr. W. E. Smith hon. secretaries.

